# The impact of basic psychological needs on academic procrastination: the sequential mediating role of anxiety and self-control

**DOI:** 10.3389/fpsyg.2025.1576619

**Published:** 2025-05-20

**Authors:** Zhaixiang Ye, Shengjie Chi, Xiaoyun Ma, Linling Pan

**Affiliations:** ^1^Department of Psychotherapy, Wenzhou Seventh People’s Hospital, Wenzhou, China; ^2^Department of Counseling, Cangnan Third Vocational School, Zhejiang, China

**Keywords:** basic psychological needs, academic procrastination, state anxiety, self-control, psychological resilience, self-determination theory

## Abstract

This cross-sectional study investigates the relationship between basic psychological needs, state anxiety, self-control, and psychological resilience in predicting academic procrastination among college students. Drawing from Self-Determination Theory, the study explores how unmet psychological needs contribute to academic procrastination through the sequential mediation of anxiety and self-control, while also examining the moderating role of psychological resilience. A sample of 612 college students participated in the study. The results reveal that basic psychological needs negatively predict academic procrastination (*β* = −0.14, *p* < 0.01) both directly and indirectly. Specifically, self-control mediates the relationship between basic psychological needs and procrastination, while anxiety and self-control serve as sequential mediators (95% CI: [−0.12, −0.06]). Furthermore, psychological resilience significantly moderates the relationship between self-control and academic procrastination (*β* = 0.08, *p* < 0.01), as well as the sequential mediation pathway. These findings underscore the critical role of psychological resilience and self-regulation in mitigating procrastination behaviors among college students, offering practical implications for educational institutions.

## 1 Introduction

In the context of ongoing higher education reform, in the context of ongoing higher education reform, Chinese universities are raising their academic standards and placing greater emphasis on talent cultivation. This process is further complicated by sociocultural expectations that prioritize institutional stability (e.g., civil service careers) and inherited traditional cultural linking academic achievement to social mobility. Among various academic challenges faced by students, academic procrastination—defined as the voluntary delay of course-related tasks despite anticipating negative consequences (e.g., task failure due to missed deadlines or compromised work quality from rushed submissions.) —has emerged as a pervasive and pressing issue (Steel, 2007). Surveys indicate that over 80% of student students engage in such behavior, with nearly 20% experiencing chronic patterns, suggesting profound implications for learning outcomes. While prior studies have established consistent links between basic psychological needs and procrastination (Asfa and Abolmaali Alhosseini, 2022; Can and Zeren, 2019; Codina et al., 2024; Oram and Rogers, 2022), the underlying mechanisms remain incompletely understood, and the cross-cultural generalizability of these findings awaits further investigation.

Self-Determination Theory (SDT) posits that effective self-regulation is contingent upon the fulfillment of three basic psychological needs: autonomy, competence, and relatedness. Some researchers conceptualize academic procrastination as a form of self-control failure. Emerging evidence suggests that unmet psychological needs may contribute to procrastination (Wang and Sun, 2023; Kurker and Surucu, 2024). Deficits in these needs are associated with heightened negative emotions, prompting individuals to seek alternative sources of fulfillment (Partala et al., 2022). This redirection of time and energy away from academic tasks toward more immediately satisfying activities is considered a core mechanism underlying procrastination.

Studies have shown that fulfilling basic psychological needs positively correlates with academic engagement and intrinsic motivation, both of which play critical roles in shaping students’ academic behaviors (Liu and Flick, 2019; Cavusoglu and Karatas, 2015). According to Self-Determination Theory, when students’ basic psychological needs are satisfied, they exhibit more autonomous motivation and goal-directed behaviors. Thus, the present study hypothesizes that the fulfillment of basic psychological needs negatively predicts academic procrastination (H1).

Anxiety can be triggered by both external and internal stimuli that an individual perceives as dangerous or threatening, with its intensity directly proportional to the perceived level of threat. Research has consistently demonstrated a negative correlation between the fulfillment of basic psychological needs and anxiety, as well as depression. When individuals are unable to satisfy these fundamental needs, they are more likely to experience heightened anxiety.

Eysenck and Calvo (1992) proposed that anxiety disrupts cognitive processes by interfering with both motivation and attention, thereby prompting individuals to engage in emotional regulation. When anxiety arises from unmet psychological needs, students may prioritize immediate emotion regulation through procrastination—a maladaptive coping strategy that temporarily reduces distress but exacerbates long-term negative consequences (Tice et al., 2001; Rahimi et al., 2023). In line with this, individuals often prioritize processing negative emotions over task completion (Tice et al., 2001). Previous studies have further established a positive correlation between anxiety and procrastination among university students, suggesting that heightened anxiety may contribute to academic delay (Fan et al., 2024). Given that unfulfilled psychological needs are associated with increased anxiety, students may postpone academic tasks as a maladaptive coping mechanism. Therefore, this study hypothesizes that anxiety mediates the relationship between basic psychological needs and academic procrastination (H2).

Self-control refers to an individual’s ability to regulate impulses, resist distractions, and override automatic responses (Baumeister et al., 2007). According to Self-Determination Theory (Deci and Ryan, 1985), the fulfillment of basic psychological needs plays a crucial role in self-control. Prior research has demonstrated that individuals who successfully meet their basic psychological needs exhibit a slower rate of self-regulatory depletion, thereby preserving greater cognitive and emotional resources for goal-directed behavior.

Baumeister et al. (2007) proposed the Strength Model of Self-Control, which posits that self-control operates as a finite resource that can be depleted over time. Students require sufficient self-regulatory resources to manage their academic behavior effectively and minimize procrastination (Choi and Yang, 2024). Given that fulfilling basic psychological needs is associated with reduced self-depletion, students who experience greater psychological need satisfaction may have more available self-control resources, thereby reducing their tendency to procrastinate. From the perspective of the Strength Model of Self-Control, need satisfaction helps conserve self-regulatory resources. Thus, this study hypothesizes that self-control mediates the relationship between basic psychological needs and academic procrastination (H3).

The fulfillment of basic psychological needs is inversely associated with anxiety. According to the self-control model, individuals regulate their negative emotions by drawing on their limited self-regulatory resources. However, within the framework of this model, such resource utilization may lead to ego depletion, which in turn impairs self-control in subsequent tasks (Baumeister et al., 2007). Individuals experiencing heightened anxiety require greater self-regulatory resources to manage their emotions, leaving fewer cognitive and attentional resources available for academic tasks (Choi and Yang, 2024). This resource reallocation makes academic procrastination more likely. Integrating both affective and cognitive pathways, anxiety may deplete self-control resources, creating a sequential mechanism. Building on this framework, the present study hypothesizes that basic psychological needs influence procrastination through the sequential mediation of anxiety and self-control (H4).

According to the strength model of self-control, depletion of self-control resources leads to a state of low self-control, increasing the likelihood of negative behaviors. This model further suggests that certain factors, particularly personality traits, can moderate the adverse effects of self-control depletion (Baumeister et al., 2007). Research has shown that positive traits, such as high moral identity and prosocial tendencies, can buffer these effects and reduce negative behaviors following resource depletion (Seeley and Gardner, 2003). Given its role in facilitating adaptive responses to adversity, psychological resilience may mitigate the impact of self-control depletion on academic behavior. Specifically, resilience is expected to attenuate the negative effects of low self-control on academic procrastination, serving as a protective factor in this process. Psychological resilience, as a protective factor, may buffer against the negative effects of low self-control. Thus, this study proposes the following hypotheses: psychological resilience moderates the relationship between self-control and academic procrastination, thereby influencing the mediating effect of self-control in the association between basic psychological needs and academic procrastination. (H5). Psychological resilience moderates the relationship between self-control and academic procrastination, further moderating the sequential mediation effect via state anxiety and self-control in the relationship between basic psychological needs and academic procrastination (H6).

## 2 Method

### 2.1 Participants

This study recruited 687 Chinese students from various universities and college using a convenience sampling method. After excluding 75 responses (10.9%) based on attentiveness checks (single-item response times ≤ 3 s; Curran, 2016), logical inconsistencies (≥2 contradictory reverse-scored items; Meade and Craig, 2012), and straightlining (≥10 identical consecutive ratings), the final sample comprised 612 participants (51.14% male, 48.86% female). The cohort reflected China’s nationally standardized tracking system: students are assigned to either (1) 4-year bachelor’s degree programs in university (47.22%, *n* = 289) if their National College Entrance Examination scores meet provincial cutoffs for academic universities, or (2) 3-year vocational diploma programs in junior college (52.78%, *n* = 323) emphasizing applied skills if scoring below this threshold. Participants represented all academic stages - 42.97% first-year (freshmen), 40.20% upper-year (including bachelor’s sophomores/juniors and vocational second-year students), and 16.83% graduating cohorts (bachelor’s seniors/vocational final-year students) - with balanced major distribution (Arts: 51.63%, Science: 18.30%, Engineering: 30.07%). This stratified composition enhances the ecological validity of findings across institutional and disciplinary contexts.

Prior to participation, all respondents were informed through a digital consent form that their involvement was strictly voluntary and could be terminated at any time without penalty. All collected data were anonymized by removing personally identifiable information and stored on secure servers with restricted access. No financial incentives were provided for participation.

### 2.2 Psychological measures

#### 2.2.1 Basic psychological needs scale (BPNS)

The Basic Psychological Needs Scale (BPNS) was developed by Deci and Ryan (2000) and later adapted for the Chinese population by Chengfu et al. (2012). This scale assesses three core dimensions—competence, autonomy, and relatedness—using 21 items rated on a seven-point Likert scale (1 = “completely inconsistent,” 7 = “completely consistent”). Nine items are reverse-scored to control for response bias. The Chinese adaptation demonstrated acceptable subscale reliabilities in the original validation: competence (*α* = 0.66), relatedness (*α* = 0.75), and autonomy (*α* = 0.70), and the internal consistency in this study was high (Cronbach’s *α* = 0.87).

#### 2.2.2 State anxiety

State anxiety was assessed using the State Anxiety Questionnaire (Xiangdong et al., 1999), which includes 20 items—11 assessing negative emotions and 9 assessing positive emotions. Participants rated each item on a four-point Likert scale, with reverse-scoring applied to positive emotion items. The scale has demonstrated high internal consistency in Chinese adaptation (*α* = 0.88), and the current sample (*α* = 0.88).

#### 2.2.3 Self-control scale (SCS)

The Self-Control Scale (SCS) developed by Tangney et al. (2004) and adapted by Shuhua and Yongyu (2008) evaluates self-regulation across five dimensions: effort, willpower, impulse control, temptation resistance, and general self-control. It contains 19 items rated on a five-point Likert scale (1 = “strongly disagree,” 5 = “strongly agree”). The Chinese adaptation (*α* = 0.86), with the current sample similarly showing high internal consistency (*α* = 0.89).

#### 2.2.4 Procrastination assessment scale for students (PASS)

Academic procrastination was assessed using the Procrastination Assessment Scale for Students (PASS), originally developed by Solomon and Rothblum (1984) and revised by Xuejing (2006). The complete PASS consists of two functionally distinct sections: (1) Part 1 (behavioral measurement) contains 18 items evaluating the frequency and severity of procrastination across six core academic tasks (e.g., “I delay starting assignments until the last minute”) using a 5-point Likert scale (1 = never to 5 = always); (2) Part 2 (causal attribution) presents a standardized scenario followed by 13 potential procrastination causes (e.g., “task aversiveness,” “perfectionism”), asking respondents to self-diagnose reasons using 5-point ratings. The current exclusively employed Part 1 because: (a) its behavioral focus directly aligns with the study’s aim to quantify procrastination levels as a dependent variable; (b) The Chinese adaptation showed similarly strong internal consistency (*α* = 0.85; Xuejing, 2006). In the current sample, reliability was excellent (*α* = 0.95).

#### 2.2.5 Connor-Davidson resilience scale (CD-RISC)

The Connor-Davidson Resilience Scale (CD-RISC), developed by Connor and Davidson (2003) and revised by Xiaonan and Jianxin (2007), was used to measure psychological resilience. The scale assesses three dimensions—hardiness, perseverance, and optimism—using a five-point Likert scale. The Chinese adaptation showed excellent internal consistency (*α* = 0.91; Xiaonan and Jianxin, 2007). In the current sample, reliability reached excellent levels (*α* = 0.95), indicating robust measurement stability.

### 2.3 Data analysis

The data were analyzed using SPSS 21.0 and Process Macro 3.5 (Hayes, 2018). Descriptive statistics and correlation analyses were conducted to examine relationships among the main variables. Multiple regression analyses and bootstrapping methods (5,000 samples) were used to test mediation and moderated mediation models. All analyses were conducted with a 95% confidence interval (CI) to determine statistical significance.

## 3 Result

### 3.1 Common method bias (CMB) test

Harman’s single-factor test was conducted to assess common method bias. The exploratory factor analysis of all items revealed 17 factors with eigenvalues greater than 1, with the first factor accounting for only 24.95% of the variance—well below the recommended threshold of 40%. This suggests that common method bias is unlikely to be a significant issue in this study (Table 1).

**Table 1:** Descriptive statistics of academic procrastination among student.

Variable	Min	Max	M	SD	Mean per item
Academic procrastination	12.0	60.0	28.89	11.74	2.41

^***^p < 0.001, ^**^p < 0.01, ^*^p < 0.05.

### 3.2 Demographic differences and influcing factors

To explore variations across demographic factors, independent samples *t*-tests and one-way ANOVA were performed, with the results presented in Tables 2, 3. Regarding academic program type, undergraduate students exhibited significantly lower levels of academic procrastination than junior college students (*t* = 10.69, *p* < 0.001). Female students reported significantly higher levels of academic procrastination than male students (*t* = −6.94, *p* < 0.001). First-year students showed significantly lower levels of procrastination than upper-year students (*p* < 0.001), while upper-year students reported lower procrastination levels than graduating students (*p* < 0.01). This trend may reflect increasing academic demands and external pressures over time. The level of academic procrastination was significantly lower among arts students and science students compared to engineering students (*p* < 0.01).

**Table 2:** Independent samples *t*-test of procrastination scores.

Variable	Category	M	SD	T
Academic program type	Vocational diploma	33.30	11.04	10.69***
	Bachelor’s degree	23.97	10.49	
Gender	Male	25.79	11.62	−6.94***
	Female	32.14	10.99	

^***^p < 0.001, ^**^p < 0.01, ^*^p < 0.05.

**Table 3:** ANOVA results for procrastination across demographics.

Variable	Category	Mean (M)	Standard deviation (SD)	*F*-value	Multiple comparisons
Major	Arts	28.54	11.83	5.92**	1 < 2**; 3 < 2**
	Science	32.18	11.43		
	Engineering	27.50	11.47		
Academic progression	First-year students	25.11	10.66	30.2***	1 < 2***; 1 < 3***; 2 < 3**
	Upper-year students	30.63	11.73		
	Graduating students	34.39	11.35		

^***^p < 0.001, ^**^p < 0.01, ^*^p < 0.05.

### 3.3 Descriptive statistics and correlation analysis of the main variables

Table 4 presents the descriptive statistics and correlation coefficients for the main variables. Basic psychological needs were positively correlated with self-control (*r* = 0.43, *p* < 0.001) and psychological resilience (*r* = 0.59, *p* < 0.001), while showing significant negative correlations with anxiety (*r* = −0.68, *p* < 0.001) and academic procrastination (*r* = −0.35, *p* < 0.001). Anxiety was also negatively correlated with self-control (*r* = −0.54, *p* < 0.001) and positively correlated with academic procrastination (*r* = 0.37, *p* < 0.001). These results indicate that students with higher satisfaction of basic psychological needs tend to have lower anxiety and procrastination levels, as well as higher self-control and resilience.

**Table 4:** Descriptive statistics and correlation analysis of main variables.

Variable	Mean	SD	1	2	3	4	5	6	7
1. Basic psychological needs	4.74	0.82	1						
2. Relatedness	5.10	0.97	0.90^***^	1					
3. Autonomy	4.50	0.84	0.88^***^	0.68^***^	1				
4. Competence	4.52	0.99	0.90^***^	0.72^***^	0.70^***^	1			
5. State anxiety	2.12	0.48	−0.68^***^	−0.54^***^	−0.63^***^	−0.66^***^	1		
6. Self-control	3.25	0.68	0.43^***^	0.34^***^	0.39^***^	0.44^***^	−0.54^***^	1	
7. Academic procrastination	2.41	0.98	−0.35^***^	−0.26^***^	−0.37^***^	−0.33^***^	0.37^***^	−0.49^***^	1
8. Psychological resilience	3.37	0.71	0.59^***^	0.47^***^	0.51^***^	0.60^***^	−0.57^***^	0.27^***^	−0.24^***^

^***^p < 0.001, ^**^p < 0.01, ^*^p < 0.05.

### 3.4 Sequential mediation effects test

Prior to testing the sequential mediation effects, we controlled for key demographic variables that may influence academic procrastination: academic program type (vocational college vs. university), academic progression (first-year to graduating cohorts), major (Arts/Science/Engineering), and gender. All study variables (basic psychological needs, state anxiety, self-control, academic procrastination, and resilience) were standardized before analysis.

A bootstrapping method (5,000 samples) was used to test the sequential mediation model (Process Model 6). The results are summarized in Table 5, indicating three significant mediation pathways:

**Table 5:** Mediation effect analysis.

Pathway	Effect size	SE	Lower limit	Upper limit
Indirect effect 1				
Basic psychological needs → State Anxiety → Academic procrastination	(−0.68) × (0.08) = −0.06	0.04	−0.13	0.02
Indirect effect 2				
Basic psychological needs → Self-Control → Academic procrastination	(0.13) × (−0.30) = −0.04	0.01	−0.07	−0.01
Indirect effect 3				
Basic psychological needs → State anxiety → Self-control → Academic procrastination	(−0.68) × (−0.45) × (−0.30) = −0.09	0.02	−0.12	−0.06
Total indirect effect	−0.06 + −0.04 + −0.09 = −0.19	0.04	−0.26	−0.11
Direct effect				
Basic psychological needs → Academic procrastination	−0.14	0.03	−0.39	−0.26
Total effect				
Indirect effect + Direct effect	−0.32	0.03	−0.39	−0.26

*Direct Effect*: Basic psychological needs directly reduced academic procrastination (*β* = −0.14, 95% CI [−0.23, −0.05]).

*Indirect Effect 1*: The mediation effect through self-control was significant (*β* = −0.04, 95% CI [−0.07, −0.01]).

*Indirect Path 2*: The sequential mediation through state anxiety and self-control was also significant (*β* = −0.09, 95% CI [−0.12, −0.06]).

The total indirect effect was −0.19 (95% CI [−0.26, −0.11]), supporting hypotheses H1, H3, and H4. However, Hypothesis H2 was not supported, as the confidence interval for the indirect effect of anxiety alone included zero (95% CI [−0.13, 0.02]).

### 3.5 Analysis of the moderating role of psychological resilience

To explore the moderating role of psychological resilience on the relationship between self-control and academic procrastination, Process Model 87 was applied while retaining the original variables. The analysis was conducted with 5,000 bootstrap iterations, and a 95% confidence interval (CI) was used to assess the significance of the moderating effect. Table 6 presents the key findings.

**Table 6:** Moderated chain intermediary test.

Dependent variable	Predictor variable	R	R^2^	F (df)	Beta	*t*	LLCI - ULCI
Academic procrastination	Basic psychological needs	0.62	0.39	42.48^***^	−0.12	−2.61^**^	−0.22 to −0.03
	State anxiety				0.04	0.87	−0.06 to 0.14
	Self-control				−0.34	−8.18^***^	−0.42 to −0.25
	Psychological resilience				−0.07	−1.70	−0.16 to 0.01
	Psychological resilience × Self-control				0.08	3.08^**^	0.03 to 0.13

^***^p < 0.001, ^**^p < 0.01, ^*^p < 0.05.

According to Table 6, basic psychological needs significantly and negatively predicted academic procrastination (*β* = −0.12, *t* = −2.61, *p* < 0.01), providing support for Hypothesis H1. Additionally, the interaction between psychological resilience and self-control was significant (*β* = 0.08, *t* = 3.08, *p* < 0.01), indicating that resilience mitigated the negative effects of low self-control on procrastination.

Further analyses demonstrated that resilience significantly moderated the simple mediation pathway and the chained mediation pathway:

- Basic psychological needs → Self-control → Academic procrastination (95% CI: [0.001, 0.024]).
- Basic psychological needs → State anxiety → Self-control → Academic procrastination (95% CI: [0.005, 0.045]).

These results provide empirical support for Hypotheses H5 and H6, confirming that psychological resilience plays a crucial role in reducing procrastination in both the direct and sequential mediation models.

### 3.6 Analysis of moderating effects via simple slope analysis

To provide a more intuitive understanding of the moderating effect of psychological resilience on the relationship between self-control and academic procrastination, a simple slope analysis was conducted. This analysis utilized the mean of psychological resilience as the reference point, with plus and minus one standard deviation representing higher and lower levels of psychological resilience, respectively.

**Figure 1:**
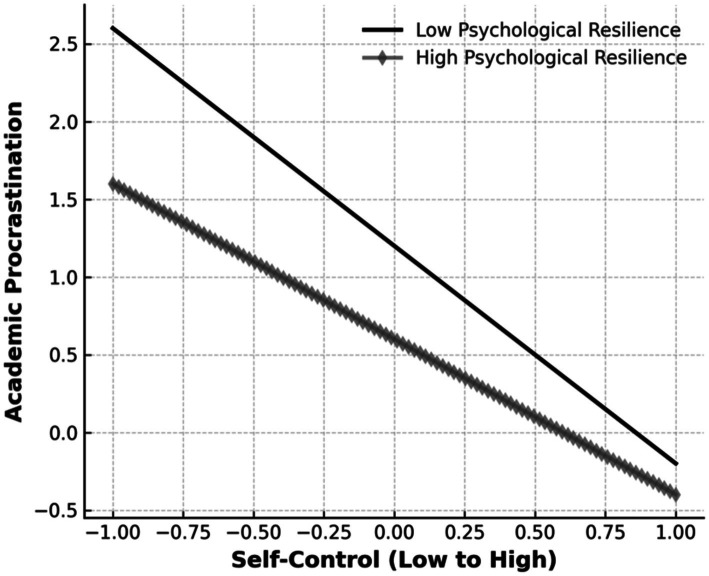
Moderating effect of psychological resilience on academic procrastination. Comparison of psychological resilience group based on self-control.

The purpose of this analysis was to investigate how self-control influences academic procrastination under varying levels of psychological resilience. The results are presented in Table 7 and Figure 1. According to Table 7, self-control showed a stronger negative association with academic procrastination at lower levels of resilience (*β* = −0.22, *p* < 0.01), compared to higher resilience levels (*β* = −0.08, *p* < 0.05). Table 8 presents the interaction effects across different resilience levels, highlighting the protective role of high resilience in buffering the adverse effects of low self-control on procrastination.

**Table 7:** Mediating effects of self-control at different levels of psychological resilience.

Psychological resilience	Effect size	Boot SE	BootCI lower limit	BootCI upper limit
−1.00	−0.05	0.02	−0.10	−0.01
0.00	−0.04	0.02	−0.08	−0.01
1.00	−0.03	0.01	−0.06	−0.01

**Table 8:** Chained mediating effects of state anxiety and self-control at different levels of psychological resilience.

Psychological resilience	Effect size	Boot SE	BootCI lower limit	BootCI upper limit
−1.00	−0.13	0.03	−0.18	−0.08
0.00	−0.10	0.02	−0.14	−0.07
1.00	−0.07	0.02	−0.11	−0.04

Figure 1 visually illustrates this interaction effect. The slope for students with high resilience is notably flatter, indicating that resilience reduces the strength of the association between self-control and procrastination. Conversely, the steep slope for students with low resilience demonstrates a stronger negative association, suggesting that students with lower resilience are more vulnerable to procrastination when their self-control resources are depleted.

In summary, the findings from the simple slope analysis align with and reinforce the results of the moderated mediation analysis. These results support the proposed hypotheses and highlight the critical role of psychological resilience in buffering the effects of self-control depletion, ultimately reducing academic procrastination.

## 4 Discussion

### 4.1 Differences in academic procrastination across demographic variables

This study analyzed the variations in academic procrastination among students across different demographic factors. Significant differences were observed based on program type, academic progression, gender, and discipline.

Students from junior college exhibited significantly higher levels of academic procrastination compared to university. This finding aligns with prior research suggesting that academic procrastination is inversely related to academic performance (Liang et al., 2021). In China, the contrasting learning environments between program type might contribute to these differences. 4-year program typically impose stricter academic requirements, which act as external motivators for students to manage their academic tasks. Conversely, the academic procrastination levels of 3-years students were found to increase year by year, possibly due to diminishing adherence to high school learning routines and an increasing focus on non-academic activities. The competitive employment environment further exacerbates procrastination, as students prioritize employability over academic engagement.

Science and engineering students demonstrated higher levels of academic procrastination than arts students, likely due to the heavier academic workload and stricter deadlines they face. The additional stress and fatigue associated with these demands may lead to procrastination as students attempt to regain emotional balance (Diotaiuti et al., 2021). The observed gender difference in procrastination—with female students reporting slightly higher levels (48.86% vs. 51.14%)—may reflect socially structured disparities rather than intrinsic traits.Female students in China face dual pressures: high academic expectations alongside persistent gender role norms (Lew et al., 2020). This conflict—between scholarly achievement and traditional social expectations—may uniquely deplete self-regulation resources, as seen in their higher procrastination levels.

### 4.2 Direct effect of basic psychological needs

The results of this study confirmed that basic psychological needs have a significant direct negative impact on academic procrastination (*β* = −0.12, *t* = −2.61, *p* < 0.01), consistent with prior findings (Mizani et al., 2015). According to self-determination theory (Deci and Ryan, 2000), the fulfillment of basic psychological needs is a fundamental driver of behavior, while unmet needs can result in negative emotions. These emotions deplete an individual’s self-control resources, reducing their capacity to complete academic tasks efficiently.

The findings further align with existing literature showing that unmet relatedness needs can lead to excessive social media use (Li, 2019; Mahboobeh et al., 2022) and that unmet competence needs often drive adolescents to engage in video games (Allen and Anderson, 2018). Such behaviors consume substantial resources, leaving students less capable of focusing on academic tasks and increasing the likelihood of academic procrastination.

### 4.3 Mediating role of state anxiety

The current study found that basic psychological needs did not significantly influence academic procrastination through the mediating role of state anxiety, with the pathway breaking down at the latter stage. This suggests that the relationship between anxiety and academic procrastination may be more complex than expectation of the current study.

First, in line with the Yerkes-Dodson law, anxiety and procrastination may exhibit an inverted U-shaped relationship (Yerkes and Dodson, 1908). Moderate levels of anxiety could initially reduce procrastination by enhancing motivation and focus, whereas excessive anxiety may impair self-regulation and lead to increased avoidance behaviors.

Second, the timing of academic tasks—particularly within China’s assessment system (which combines continuous evaluations and high-stakes final exams)—likely plays a moderating role. We propose that: early phases (distant deadlines): Anxiety may trigger emotion-focused coping, leading to procrastination when perceived consequences are minimal, late phases (approaching deadlines): As the costs of delay become salient, students may override anxiety-driven avoidance and engage with tasks.

This temporal perspective helps explain why a straightforward mediation model was unsupported, highlighting the need for future research to examine anxiety-procrastination dynamics across different academic phases.

### 4.4 Mediating role of self-control

This study validated the hypothesis that basic psychological needs influence academic procrastination through the mediating role of self-control. The findings indicate that individuals with fulfilled psychological needs tend to exhibit higher levels of self-control, consistent with the strength model of self-control theory (Baumeister et al., 2007).

From a theoretical perspective, unmet basic psychological needs often generate negative emotions, which require self-control resources to regulate (Ryan and Deci, 2019). This emotional regulation depletes the resources needed for academic tasks, increasing the likelihood of procrastination. Conversely, the fulfillment of basic psychological needs promotes positive emotions, which have been shown to replenish self-control resources (Orkibi and Ronen, 2017; Kurker and Surucu, 2024). Therefore, students with higher self-control are better equipped to manage their academic responsibilities and are less likely to procrastinate.

### 4.5 Chain mediating effect of state anxiety and self-control

The study further revealed that basic psychological needs influence academic procrastination through the chain mediating effect of state anxiety and self-control. Individuals with unmet psychological needs are more prone to experiencing anxiety in response to life events such as interpersonal conflicts and academic challenges (Orkibi and Ronen, 2017; Kurker and Surucu, 2024). Anxiety exacerbates self-control resource depletion, as individuals must regulate their heightened emotional states, leaving fewer resources for academic tasks.

Additionally, anxiety is known to interfere with attention, making it more challenging to concentrate on academic work (Eysenck and Calvo, 1992). Poor sleep quality, often associated with high anxiety levels, further hinders the recovery of self-control resources (Garcia and Cooper, 2024). Consequently, individuals experiencing high anxiety are at greater risk of self-control failure, which ultimately leads to increased academic procrastination.

### 4.6 Moderating role of psychological resilience

The study confirmed the moderating role of psychological resilience in the relationship between self-control and academic procrastination, as well as its influence on the chain mediating pathway (basic psychological needs → state anxiety → self-control → academic procrastination). According to the strength model of self-control, personality traits such as resilience can buffer the negative effects of self-control resource depletion (Baumeister, 2002). Students with high psychological resilience are more capable of enduring challenges and persisting in academic tasks, even when self-control resources are limited. Resilience enables individuals to maintain focus and effort, thereby reducing the impact of resource deficits on academic procrastination. While psychological resilience does not directly influence academic procrastination, it serves as a significant protective factor by mitigating the adverse effects of low self-control.

## 5 Conclusion

This study investigated the effects of basic psychological needs, state anxiety, self-control, and psychological resilience on academic procrastination among college students. The findings yielded the following key conclusions:

(1) Academic procrastination exhibits significant differences across demographic factors, including educational attainment, grade, gender, and discipline.
(2) Basic psychological needs negatively predict academic procrastination, both directly and indirectly through the mediating role of self-control and the chain mediating effect of state anxiety and self-control.
(3) Psychological resilience significantly moderates the relationship between self-control and academic procrastination, buffering the adverse effects of self-control depletion.

### 5.1 Theoretical contributions

From a theoretical perspective, this study extends self-determination theory by enriching research on academic procrastination and broadening the framework of basic psychological needs theory. Furthermore, by incorporating psychological resilience into the model and utilizing a Chinese college student sample, this study provides cross-cultural validation for the role of personality traits in moderating the consequences of ego depletion.

### 5.2 Practical implications

In terms of campus environment, universities should emphasize the role of student organizations and foster a supportive social atmosphere to meet students’ psychological needs and alleviate academic anxiety. Additionally, from an institutional perspective, universities should:

Strengthen the development of psychological counseling centers.

Implement group counseling programs and mental health courses tailored to students at different academic levels.

Design targeted interventions to help students develop self-regulation strategies and mitigate academic procrastination.

### 5.3 Limitations and future directions

Despite its contributions, this study has several limitations that warrant discussion.The cross-sectional design, while efficient for preliminary model testing, does not capture longitudinal data across students’ stress cycles (e.g., semester start to final exams). For the theoretical model examined in this study, temporal factors may constitute an important latent moderator.

Methodological constraints should also be acknowledged. The reliance on convenience sampling from participating universities, while pragmatically necessary for data collection efficiency, may limit the generalizability of findings to broader student populations. This approach inherently excludes non-responsive student subgroups (e.g., those less engaged with online surveys). The reliance on online questionnaires, though cost-effective, may affect data representativeness, particularly for populations with limited internet access. Furthermore, self-report measures introduce the potential for response bias, as evidenced by slight gender differences in reported procrastination patterns (males 51.14% vs. females 48.86%). Future studies could integrate objective behavioral metrics, such as learning management system (LMS) activity logs, to complement self-reported data.

The sample characteristics present notable limitations in demographic representativeness. Although the institutional composition (junior college: 52.78%; undergraduate: 47.22%) reflects educational diversity, the disproportionate concentration of arts majors (51.63%) compared to science (18.30%) and engineering (30.07%) disciplines may skew findings, particularly given established field-specific differences in procrastination behaviors (e.g., deadline structures in studio-based vs. lab-based coursework). Stratification by academic stage further reveals imbalance: 42.97% first-year students, 40.20% upper-year cohorts, and only 16.83% graduating students—a critical gap as final-year populations face unique thesis/project deadlines. The near absence of part-time (0%) and limited vocational students in the sample exacerbates generalizability concerns. These limitations interact with cultural context; China’s exam-centric system likely intensifies anxiety effects, particularly for first-years (gaokao transition) and graduates (employment pressures).

To address these issues, future research could pursue three key directions: First, longitudinal designs tracking need satisfaction, anxiety fluctuations, and procrastination behaviors across semesters could clarify causal sequences. Second, targeted interventions (e.g., autonomy-supportive training for instructors) could experimentally test the model’s practical utility. Finally, cross-cultural replications would evaluate the universality of these mechanisms. Future studies could also employ diversified sampling strategies to avoid over-concentration in particular student subgroups.

In conclusion, while these limitations caution against overgeneralization, the study’s novel integration of sequential mediation and moderation analyses advances theoretical understanding and provides a foundation for evidence-based interventions in academic settings.

## Data Availability

The raw data supporting the conclusions of this article will be made available by the authors, without undue reservation.
